# Sequential expression of putative stem cell markers in gastric carcinogenesis

**DOI:** 10.1038/bjc.2011.287

**Published:** 2011-08-09

**Authors:** T Wang, C W Ong, J Shi, S Srivastava, B Yan, C L Cheng, W P Yong, S L Chan, K G Yeoh, B Iacopetta, M Salto-Tellez, Singapore Gastric Cancer Consortium

**Affiliations:** 1 Cancer Science Institute, National University Health System and Yong Loo Lin School of Medicine, National University of Singapore, Singapore; 2 Department of Pathology, National University Health System and Yong Loo Lin School of Medicine, National University of Singapore, Singapore; 3 School of Surgery, University of Western Australia, Perth, Australia

**Keywords:** CD44, CD133, Musashi-1, Correa pathway, gastric cancer

## Abstract

**Background:**

Gastric carcinogenesis has been well documented in the step-wise histopathological model, known as Correa pathway. Several biomarkers including CD44, Musashi-1 and CD133 have been reported as putative stem cell (PSC) markers.

**Methods:**

We investigated expression of PSC markers CD44, Musashi-1 and CD133 in relation to gastric carcinogenesis and prognosis and chemoresponse. Immunohistochemistry staining was performed in gastric cancer (GC) clinical specimens representing different steps of the Correa pathway. Gastric cancer samples taken before and after neoadjuvant chemotherapy with docetaxel, cisplatin and capecitabine (DCX) were also evaluated for PSC marker expression.

**Results:**

We showed that the expression of three PSC markers was significantly elevated in GC relative to normal gastric mucosa (*P*<0.001 for each marker). Precancerous lesions, including intestinal metaplasia and dysplasia, demonstrated increased expression of CD44 and Musashi-1. CD133 was predominantly expressed along the border between intramucosal carcinoma and connective tissue at later stages. High CD44 and CD133 expression showed prognostic value for worse patient survival (*P*=0.014 and *P*=0.019, respectively). A small number of tumours with high expression of CD44 and CD133 showed pathological response to DCX-based neoadjuvant chemotherapy.

**Conclusion:**

CD44 and Musashi-1 are frequently expressed in both premalignant gastric lesions and invasive GC, whereas CD133 expression is restricted mainly to neoplastic tissues.

Gastric cancer (GC) is the second most common cause of cancer-related mortality worldwide. It often arises in the context of chronic inflammation induced mainly by *Helicobacter pylori* (*Hp*) (Konturek *et al*, 1999). The inflammatory microenvironment, sometimes referred to as an altered stem cell niche, favours the promotion of cancer and can also affect cancer initiation and progression. Changes to the stem cell niche might be responsible for the transformation of stem or progenitor cells to cancer stem cells (CSC) via the acquisition of various genetic and epigenetic events (Gonda *et al*, 2009). Cancer stem cells are defined as having the capacity for self-renewal and giving rise to the heterogeneous lineages of cancer cells that eventually constitute the tumour (Clarke *et al*, 2006). There is increasing evidence to suggest that CSC are highly resistant to chemotherapy and may be responsible for tumour recurrence following chemotherapeutic intervention (Klonisch *et al*, 2008). Gastric CSC could arise from transformed adult tissue-specific stem cells located within the gastric isthmus/neck of corpus glands and at the base of antrum glands. Alternatively, they could arise from circulating bone marrow-derived stem cells (BMDSCs). These cells have been shown to contribute to tumour formation in animal models and possibly also in humans with *Hp*-induced chronic gastric inflammation (Li *et al*, 2006).

In general, there is an expectation that molecules expressed by normal stem cells can serve as markers of CSC. Several biomarkers including CD44 (Takaishi *et al*, 2009), Musashi-1 (Potten *et al*, 2003; Gotte *et al*, 2008) and CD133 (Smith *et al*, 2008; McCord *et al*, 2009) have been reported as putative stem cell (PSC) or CSC markers. CD44 is involved in cell–cell and cell–matrix interactions (Gulmann *et al*, 2003) and has been used as a CSC marker in leukaemia and cancers of the pancreas, breast, prostate and head and neck (Klonisch *et al*, 2008). CD44+ sub-population of GC cell lines (Takaishi *et al*, 2009) shows characteristics of tumour-initiating cells. CD44^high^ gastrointestinal tumours have increased defence against reactive oxygen species (ROS) by enhancing capacity for reduced glutathione synthesis and thereby promote cancer cell proliferation (Ishimoto *et al*, 2011). CD44 expression is also a marker for the progression and metastasis of GC (Castella *et al*, 1998; Yoo *et al*, 1999; Ghaffarzadehgan *et al*, 2008). The Musashi-1 gene encodes an RNA-binding protein that has been proposed as a PSC marker in the mouse intestine and human stomach (Kayahara *et al*, 2003; Potten *et al*, 2003; Akasaka *et al*, 2005). Musashi-1 facilitates malignant transformation through multiple acquired chromosomal aberrations (Siebzehnrubl *et al*, 2009) and is a CSC marker in tumours (Hemmings, 2010). Finally, CD133 is a cell surface glycoprotein that has been widely used to identify stem cell from normal and cancerous tissues (Wu and Wu, 2009). It has also been proposed as a therapeutic target in GC (Smith *et al*, 2008).

The link between chronic gastritis and carcinogenesis has been well documented in the step-wise histopathological model of gastric carcinogenesis proposed by Correa (1988). This model includes the morphologically defined steps of chronic gastritis, intestinal metaplasia (IM), dysplasia and invasive tumour. Intestinal metaplasia is an important premalignant lesion in the Correa pathway and is present in >80% of intestinal type GC cases (Conchillo *et al*, 2001). Although McDonald *et al* (2008) demonstrated that both gastric and intestinal metaplastic crypts were clonal, contained multipotential stem cells, little is known about the role of PSC markers in the overall Correa pathway of gastric carcinogenesis. The evaluation of PSC marker expression along this pathway could help determine the extent to which GC development relies on the stem cell compartment. Moreover, a better understanding of the associations between PSC marker expression and the prognosis and chemoresponsiveness of GC could help in the development of more effective therapies directed against cells with ‘stem-like’ properties. The aims of this work were therefore to evaluate the expression of CD44, Musashi-1 and CD133 at different steps in the Correa pathway of GC and to determine their prognostic and predictive significance in this cancer type.

## Materials and methods

### Clinical samples and clinicopathological data

A total of 116 GC cases that were surgically resected at the National University Hospital of Singapore (NUHS) between 2000 and 2004 were included in construction of the first tissue microarray (TMA1) as described below. Among these, 10 cases of intestinal type GC taken from the transition area between malignancy and adjacent normal mucosa were also selected for full section analysis. Histologically, these cases showed pathological steps along the Correa pathway. An additional cohort of 38 cases with chronic gastritis, IM and intestinal type GC was selected for the construction of a second TMA (TMA2). Finally, five cases with complete type IM and five cases with incomplete IM were selected as well.

Clinicopathological characteristics of the study cohort are summarised in Supplementary Table S1. The available clinicopathological information included age, ethnicity, sex, tumour stage (TNM), tumour grade, histological type, *Hp* co-infection, perineural invasion, lymphatic invasion and cancer-specific survival. *Helicobacter pylori* co-infection was defined as the microscopic presence of scattered or colonising *Hp* along the gastric mucosa surface or gastric pits. Overall survival was defined as the interval between surgery and death or the last observation taken. The data were censored at the last follow-up period for living patients. Staging was based on pathological findings according to the American Joint Committee on Cancer classification (Greene, 2002).

To assess the predictive value of PSC markers for response to chemotherapy, eight patients with local GC (stage II or III) were recruited from the NUHS phase II trial of docetaxel, cisplatin and capecitabine (DCX; GA02/33/06). The clinicopathological information for this cohort is shown in Supplementary Table S2. Tumour specimens were obtained before and after neoadjuvant chemotherapy by means of endoscopic biopsy and surgical gastrectomy, respectively. All patients were treated with three cycles of docetaxel (30 mg m^−2^), cisplatin (30 mg m^−2^) and capecitabine (750 mg m^−2^). Pathological response to neoadjuvant chemotherapy was defined as the absence of tumour or only foci of residual invasive tumours. Multiple slides were evaluated to assess pathological response, as described earlier (Becker *et al*, 2003).

### Construction of TMAs

Tissue microarray blocks containing cores from 116 primary GC cases (TMA1) were constructed as described previously (Salto-Tellez *et al*, 2007). Briefly, a donor core from a morphologically representative area of donor tissue block was punched using a 1 mm diameter needle. The core was subsequently inserted into a recipient paraffin block using an ATA-100 tissue arrayer (Chemicon, Temecula, CA, USA). Cores were taken from the tumour mass (three cores) and from matched, histologically normal gastric mucosa (three cores) for each case. Tissue microarray-2 was created in the same manner as TMA1 and comprised samples of chronic gastritis, IM and intestinal type GC tissues.

### Immunohistochemical analysis

Consecutive sections (4 *μ*m thickness) were cut from TMAs and placed on slides for IHC analysis. Full sections (10 cases of intestinal type GC, 10 cases of IM and 8 pairs of biopsies before and after chemotherapy) were also cut for IHC. Antigen retrieval was carried out with 10 mM citrate buffer (pH 6.0) in a MicroMED TT Microwave Processor (Milestone, Sorisole, Italy) for 5 min at 120 °C. The slides were then incubated with primary antibodies against CD44, Musashi-1, CD133, Ki67 and proliferating cell nuclear antigen (PCNA) for 12 h at the dilutions indicated in Supplementary Table S3. Information on the antibody clone and the commercial supplier are also shown in Supplementary Table S3. Immunostaining was performed with the streptavidin-biotin kit (LSAB2, Dako, Oslo, Norway) in accordance with the manufacturer's instruction and the slides were then counterstained with haematoxylin. For CD44, inflammatory cells in the stroma of GC served as internal positive control (Sneath and Mangham, 1998). A known colorectal cancer from one of our previous studies was the positive control for CD133 (Ong *et al*, 2010). A 12-week gestation fetal brain was used as positive control for Musashi-1 (Chan *et al*, 2006). Negative controls consisted of omission of the primary antibody without any other changes to subsequent procedures. For histological investigation, sections were stained with haematoxylin and eosin.

### IHC scoring

The evaluation of immunostaining was performed independently by two gastrointestinal pathologists (TW and SS), both of whom were blinded to the clinicopathological data, and supervised by a third, senior pathologist (MST). Expression of the three PSC markers on epithelial cells from TMA1 and full sections was scored by multiplying the intensity and extent of staining from the same core according to the formula: 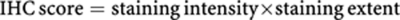


The range was from 0 to 300. Staining intensity was classified into four groups: 0 (negative), 1 (weak), 2 (moderate) and 3 (strong). Staining extent was defined as the percentage of positive staining cells in the total cancer compartment. The cut-off scores for determining positive expression for each marker were determined by receiver-operating characteristic (ROC) curve analysis as outlined previously (Zlobec *et al*, 2007). For ROC analysis, scores above the cutoff were considered positive for protein expression and scores below as negative. Receiver-operating characteristic analysis also allows the identification of markers that possess discriminatory value for the determination of prognostic significance through the area under ROC curve values. Distribution of PSC marker expression along the Correa pathway was evaluated according to the number of CD44, Musashi-1 and CD133 positive biopsies in TMA2. Tumours with >20% of PCNA immunostaining in cancer cells was defined as positive cases (Li *et al*, 2008a).

### High-iron diamine-Alcian blue (HID-AB) staining

Full sections from IM were carried out for HID-AB staining according to a standard pathology diagnostic protocol. Briefly, sections were treated in a HID solution overnight and subsequently incubated with 1% AB at pH2.5 for 5 min.

### Determination of subtype IM

The discrimination of complete *vs* incomplete types of gastric IM was assessed on the basis of the morphology on H&E- and HID-AB-stained sections as described previously (Correa *et al*, 2010). Complete type IM was characterised by epithelium resembling the small intestinal phenotype: a well-defined brush border and sialomucins in goblet cells by HID-AB staining; incomplete type IM resembled the colonic epithelium phenotype: variable size mucin droplets in the cytoplasm, absence of a brush border and HID-AB showing sialomucins in goblet cells and a mixture of neutral and sulfomucins in columnar cells.

### Statistical analysis

Differential expression of PSC markers between tumour and matched normal mucosa or between preoperative and gastrectomised tumour was analysed using the *t*-test. The co-relationships between markers and their associations with clinicopathological variables were determined using the Pearson correlation coefficient and *χ*^2^-test, respectively. Survival analysis was performed with the Kaplan–Meier method and the log-rank test was used to compare groups. The independence of prognostic factors for survival was evaluated by the multivariate Cox regression model using a stepwise selection procedure. The covariates included in the multivariate Cox regression model were age (⩾64 *vs* <64 years), tumour stage (III–IV *vs* I–II), grade (poor-undifferentiated *vs* well-moderate), histological type (intestinal *vs* diffuse), lymphatic invasion (yes *vs* no), perineural invasion (yes *vs* no), CD44 expression (positive *vs* negative), Musashi-1 (positive *vs* negative) and CD133 expression (positive *vs* negative). All statistical analyses were performed using the SPSS package (version 15.0 for Windows, SPSS Inc., Chicago, IL, USA) with significance set at the 5% level.

## Results

### PSC marker expression in normal gastric mucosa and in GC

**Figure 1:**
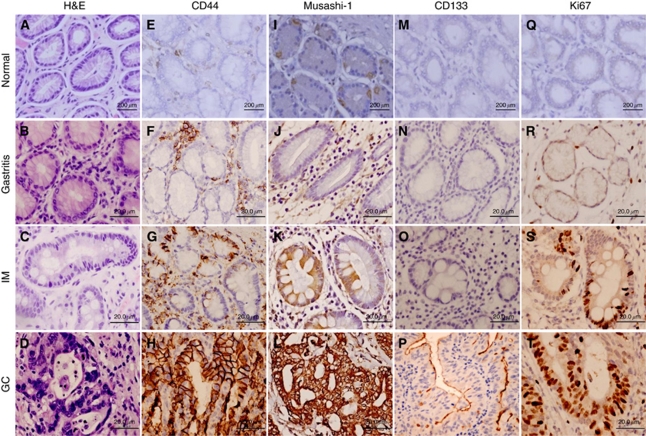
Putative stem cell marker expression during the Correa pathway of gastric carcinogenesis. Representative images of normal, gastritis, intestinal metaplasia (IM) and malignant (GC) epithelial tissues from gastric specimens are shown following immunohistochemical staining for CD44 (**E**–**H**), Musashi-1 (**I**–**L**) and CD133 (**M**–**P**). Representative haematoxylin and eosin (H&E) stains (**A**–**D**) and immunohistochemical staining for Ki67 (**Q**–**T**) are also shown.

The expression of PSC markers in gastric tissue samples was investigated by IHC. CD44 and Musashi-1 showed consistent expression pattern in both intestinal type and diffuse type cancer, with respect to their localisation in the adjacent normal area and cancer. CD44 expression was predominantly localised to the membrane of tumour epithelial cells (Figure 1H). The strong concomitant staining was seen in many inflammatory cells located in the stroma. CD44 expression was absent in the majority of normal gastric mucosa samples (Figure 1E). Staining for Musashi-1 was predominantly located in the cytoplasm of tumour cells (Figure 1L), with occasional nuclear staining. Mild staining for Musashi-1 was also observed in the lower third of gastric body mucosa in a minority of cases (Figure 1I). In line with the expression pattern reported in other studies (Ishigami *et al*, 2010; Ong *et al*, 2010), staining for CD133 was observed at the luminal surface of tumour cells in intestinal type GC (Figure 1P). In diffuse type GC, our result showed CD133 expression was localised to the cytoplasm. No CD133 expression was observed in normal gastric mucosa taken from the gastric body or antrum (Figure 1M).

Using the scoring criteria outlined in the Materials and Methods section, the frequency of positive expression for each marker is shown in Table 1. Using values derived from the area under the ROC curve, markers with area under ROC curve values above 0.5 indicate significant discriminatory power for survival. Musashi-1 and CD133 were identified as having discriminatory power (area under the ROC curve value >0.5). Positive expression for CD44, Musashi-1 and CD133 was observed in 77% (82 out of 106), 85% (87 out of 102) and 44% (45 out of 103) of primary GC samples from TMA1 (Table 1), respectively. PSC marker expression was significantly upregulated in GC compared with the matching normal gastric mucosa for each case (Table 2, *P*<0.001 for each marker). The positive rate for PCNA was 85% (83 out of 98) in TMA1.

CD44 expression was weakly associated with Musashi-1 expression (*r*=0.265, *P*=0.006). The expression of CD133 was not associated with the expression of either CD44 or Musashi-1. Both CD44 and Musashi-1 were weakly associated with the proliferation marker PCNA (*r*=0.248, *P*=0.014 and *r*=0.240, *P*=0.024, respectively).

### PSC marker expression during the Correa pathway

The approximate extent and topology of PSC marker expression along the Correa pathway of gastric carcinogenesis was studied using TMA2 containing representative tissue samples with gastritis, IM and intestinal type GC (Figure 1). Both the proportion of positive cells and the topological distribution of expression showed considerable variability and ranged from complete lack of immunoreactivity (Figures 1J, N and O), expression in single cells (Figures 1G and K) and staining of cell clusters (Figures 1H, L and P).

**Figure 2:**
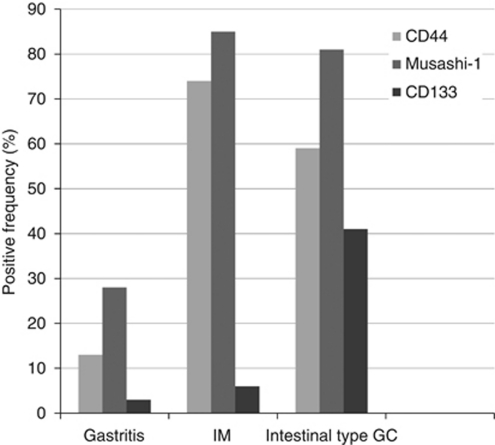
Frequency of positive expression for PSC markers along the Correa pathway. Values were determined following immunohistochemical staining and scoring as described in the Materials and Methods section.

The frequency of positive expression for the PSC markers is shown in Figure 2 for the gastritis, IM and intestinal type GC tissue samples from TMA2. CD44 was not detectable in 87% (26 out of 30) of gastritis cases, while was positive in 73% (14 out of 19) of IM and 59% of intestinal type GC (17 out of 27). Musashi-1 was expressed in 10 out of 36 cases of gastritis (28%), 17 out of 20 of IM (85%) and 30 out of 37 of intestinal type GC (81%). In contrast, CD133 expression was found in 1 out of 31 cases of gastritis (3%), 1 out of 18 cases of IM (6%) and 11 out of 27 cases of GC (41%). Taken together, the expression of all three markers was lower in gastritis compared with the later stages. Intestinal metaplasia and intestinal type GC showed comparable frequencies of CD44 and Musashi-1 expression, but CD133 expression was considerably more frequent in intestinal type GC.

To further explore the distribution pattern of CD44 and Musashi-1 expression in different types of IM, IHC was carried out on 10 full sections from cases with either complete type IM or incomplete type IM (5 each). CD44 was observed in 80% (4 out of 5) of both complete IM and incomplete IM whereas Musashi-1 was seen in 100% (5 out of 5) of both complete IM and incomplete IM. Therefore, with respect to the frequency of CD44 and Musashi-1 expression, there was no obvious difference between complete type IM and incomplete type IM.

Immunostaining for Ki67 revealed that IM and intestinal type GC glands contained a higher proportion of proliferating cells (Figures 1S and T) compared with normal or gastritis epithelium (Figures 1Q and R). This increased cell proliferation coincided with an increase in the expression of CD44 and Musashi-1 during progression from the normal/gastritis to IM/GC stages (Figure 2).

**Figure 3:**
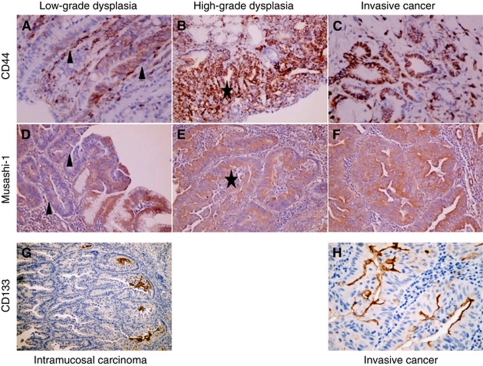
Expression of PSC markers as revealed by immunohistochemical staining on full-face sections of intestinal type GC. Representative images of CD44 and Musashi-1 expression in low grade dysplasia (**A** and **D** (arrows)), high grade dysplasia (**B** and **E** (stars)) and invasive cancer (**C** and **F**), as well as CD133 in intramucosal carcinoma (**G**) and invasive cancer (**H**).

IHC staining for CD44, Musashi-1, CD133 and Ki67 was also performed on 10 cases of full-face sections containing the characteristic morphological stages of the Correa pathway (Figure 3). CD44 and Musashi-1 expression were common in lesions showing low (Figures 3A and D) and high levels of dysplasia (Figures 3B and E), but not CD133 expression. CD133 immunostaining was especially prominent at the border between neoplastic epithelium and connective stromal tissue (Figure 3G), supporting the notion that it may be linked to tumour cell migration (Ishigami *et al*, 2010). No difference in the staining intensity of these markers was apparent between high-grade dysplasia and invasive cancer (CD44 and Musashi-1), nor between intramucosal carcinoma and invasive cancer (CD133; Figures 3G and H).

### Expression of PSC markers and clinicopathological features of GC

The expression of PSC markers was evaluated in relation to standard clinicopathological variables for GC (Supplementary Table S1). CD44 expression was significantly more frequent in GC with poor/undifferentiated compared with well/moderate differentiation (*P*=0.027) as well as in GC with diffuse type compared with intestinal type histology (*P*=0.016). Higher expression of Musashi-1 was associated with advanced T-stage and TNM stage (*P*=0.007 and *P*=0.047, respectively). CD133 expression was not associated with any of the clinicopathological variables examined here.

### Prognostic significance of PSC marker expression in GC

**Figure 4:**
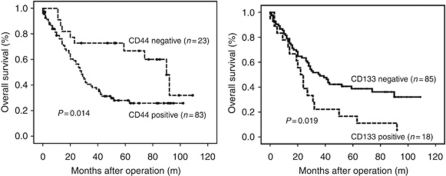
Kaplan–Meier survival analysis of GC patients according to expression levels for the PSC markers CD44 and CD133.

Positive CD44 and CD133 expression were associated with worse overall survival (Figure 4; *P*<0.05 for each). Musashi-1 expression was not associated with survival. Multivariate Cox regression analysis showed that patient age ⩾64 years (HR=1.05; 95% CI: 1.02–1.08) and advanced tumour stage (HR=3.31; 95% CI: 2.33–4.72) were independent prognostic markers for overall survival. None of the PSC markers showed independent prognostic value.

### Predictive significance of PSC marker expression for response to neoadjuvant chemotherapy

**Figure 5:**
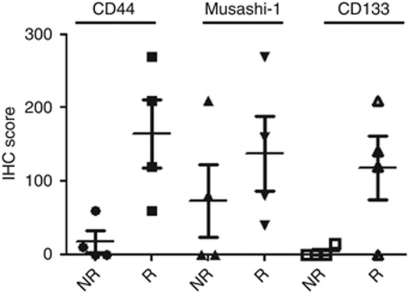
Expression of PSC markers in pretreatment biopsies of GC. Four tumours showed pathological response to treatment (R) and four tumours did not (NR). A significant difference between R and NR tumours was observed for the average expression level of CD44 and CD133 (*P*<0.05 for each).

**Figure 6:**
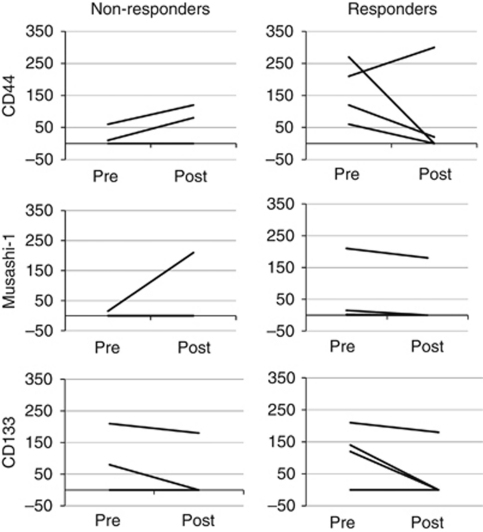
Changes in the expression of PSC markers following neoadjuvant chemotherapy of GC. Tumours showing pathological response are shown separately to those in which no response was observed.

A pathological response was observed in 50% (4 out of 8) of patients who received DCX-based neoadjuvant chemotherapy. Significantly more expression of CD44 and CD133 was observed in preoperative biopsies from responsive compared with non-responsive cases (*P*=0.023 and *P*=0.041, respectively; Figure 5), but no significant difference was seen for Musashi-1 expression. These results should be interpreted with caution, however, because the limitation of small sample size. Changes in expression of the three PSC markers following DCX-based neoadjuvant chemotherapy are shown in Figure 6 for each tumour. The expression of each marker decreased in the majority of tumours showing pathological response to chemotherapy, but this was not apparent for non-responsive tumours.

## Discussion

**Figure 7:**
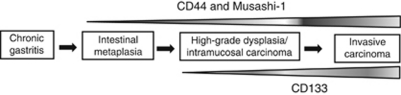
Schematic representation of PSC marker expression along the Correa pathway. The expression of CD44 and Musashi-1 is frequently observed in IM, dysplasia and invasive cancer stages, whereas CD133 expression is observed at the intramucosal carcinoma and invasive cancer stages.

Chronic gastritis promotes the proliferation of gastric adult stem cells and also leads to the recruitment of BMDSCs into the gastric mucosa, both of which may contribute to tumour development (Gonda *et al*, 2009). In the present work, we provide the first histological link between the expression of three PSC markers (CD44, Musashi-1 and CD133) and gastric carcinogenesis as characterised by the Correa pathway. A schematic representation of the expression of these markers along the Correa pathway is proposed in Figure 7. We also investigate the expression of PSC markers in relation to the clinical outcome of GC (Figure 4) and the response to chemotherapy (Figure 5).

Previous studies have demonstrated that a synergy between inflammation and host factors is required for effective gastric carcinogenesis to occur (Figueiredo *et al*, 2002). Chronic gastritis, which elicits the activation of an adaptive immune response (T and B cells), contributes substantially to development of the characteristic histological features in the Correa pathway (D’Elios *et al*, 2005). The morphologically identifiable precancerous lesions along this pathway are thought to represent the steps by which intestinal type GC initiates and progresses. Among these, IM represents the transition of normal gastric mucosa to an intestinal phenotype that expands through monoclonal conversion of multipotential stem cells (McDonald *et al*, 2008). Therefore, IM formation in the background of chronic gastritis may result from mutated gastric stem cells that undergo intestinal-type crypt transformation. Microarray-based gene expression profiling and IHC staining have shown increased expression of putative gastric progenitor cell markers in IM, including villin (Boussioutas *et al*, 2003; Qiao *et al*, 2007) and CD44 splice variant-6 (Gulmann *et al*, 2003).

Our data support the above hypothesis for IM formation by showing increased expression of the intestinal stem cell markers CD44 and Musashi-1 in IM relative to gastritis (Figure 2), suggesting these may have an important role in the malignant transformation of IM. Thus, CD44 and Musashi-1 may be useful as diagnostic markers for the detection of precursor lesions such as IM and dysplasia, as well as for the prediction of cancer risk in patients with IM in GC. Furthermore, our results showed co-expression of CD44 and Musashi-1 (*r*=0.265, *P*<0.05), similar to the co-expression of these PSC markers reported in colorectal cancer (Schulenburg *et al*, 2007). Taken together, the expression of CD44 and Musashi-1 in the IM and dysplastic precancerous lesions suggests these may be early events in gastric carcinogenesis that contribute to the initiation of GC. Their frequent expression in invasive cancer (Figure 2) also suggests they have a role in the progression of GC. A recent study showed that the association between a CD44 variant and glutamate–cystine transporter blocked ROS-induced stress signalling, promoting the proliferation of gastrointestinal CSC, which expressed high levels of CD44 (Ishimoto *et al*, 2011). Moreover, *Hp*, either directly or through the induction of a local inflammatory response, may be responsible for the increased expression of CD44 (Fan *et al*, 1996) and Musashi-1 (Murata *et al*, 2008), suggesting a possible link between CD44/Musashi-1 expression and chronic gastritis. Although we did not observe an association between *Hp* infection and the expression of PSC markers in the present study, this could be due to the small sample size of *Hp-*positive cases detected (*n*=28). *Helicobacter pylori* diagnosis in this study was based on concomitant infection, but not past-infection or marked infection since intestinal metaplastic changes may eliminate *Hp* (Konturek *et al*, 2009).

The lack of CD133 immunostaining in gastric precursor lesions (Figure 2) suggests this protein contributes to gastric carcinogenesis in a manner that is different to CD44 and Musashi-1. A previous study showed that putative BMDSCs identified using CD133 (Yin *et al*, 1997) may also have an important role in the development of GC (Gonda *et al*, 2009). Futagami *et al* (2010) demonstrated that CD133-positive cells could migrate to the bottom of gastric epithelium in *Hp*-infected gastritis and GC tissues in the Mongolian gerbil animal model. Intramucosal carcinomas may secrete chemotactic cytokines such as FGF2 and VEGF (Ritter *et al*, 2008) that trigger the homing of circulating BMDSC to the tumour mass, thereby contributing to tumour growth via mesenchymal–epithelial transformation. Furthermore, CD133 expression may also be upregulated through the inhibition of mTOR signalling (Matsumoto *et al*, 2009) to promote cancer cell metastasis into distant organs (Al Dhaybi *et al*, 2010; Shimada *et al*, 2010). This may explain the worse survival of GC patients with high CD133 expression observed in the current work (Figure 5) and in a previous study (Ishigami *et al*, 2010). CD133 expression, therefore, appears to be a later event in gastric carcinogenesis compared with the expression of CD44 and Musashi-1 (Figure 7).

The CSC hypothesis suggests these cells have chemoresistant properties and this subsequently leads to tumour recurrence and metastasis. The evidence of the effect of conventional chemotherapy on the stem cell population is contradictory: while some studies showed it to be ineffective against CSC as it generally increases the percentage of stem cell population (Li *et al*, 2008b; Creighton *et al*, 2009), other studies demonstrated that chemotherapy reduced the number of breast and colon CSC using CD44+/CD24− or CD133 as CSC marker respectively (Aulmann *et al*, 2010; Hongo *et al*, 2011). Our preliminary results obtained in a small cohort (*n*=8) of neoadjuvant treated GC patients also challenged this notion (Figure 5). A small number of tumours with high CD44 or CD133 expression in pretreatment biopsies nevertheless showed good pathological response to DCX-based chemotherapy. Furthermore, the expression of CD44 and CD133 decreased following chemotherapy in the majority of responsive tumours (Figure 6). The induction of terminal differentiation (Beug, 2009) may explain the decrease in expression of PSC markers following chemotherapy in this small group of tumours. Additional studies in larger cohorts are required, however, to clarify the predictive significance, if any, of the expression of PSC markers in GC.

In conclusion, this is the first study to investigate the expression patterns and prognostic and predictive significance of a panel of PSC markers in GC. In the process of gastric carcinogenesis, the expression of CD44 and Musashi-1 appear to be early events and to remain high in later stages. The increase in CD133 expression occurs later and is found predominately at the edge of intramucosal carcinoma, suggesting it has a role in tumour extension. High CD44 and CD133 expression were associated with worse prognosis, but more studies are required to establish the predictive value of PSC markers.

## Figures and Tables

**Table 1:** Frequency of positive expression of PSC markers using cut-off scores derived from the area under the ROC curve

**PSC markers**	**Area under ROC**	**Cut-off score**	**Positive expression *n* (%)**
CD44	0.501	5	82 (77)
Musashi-1	0.568	150	87 (85)
CD133	0.523	50	45 (44)

Abbreviations: PSC=putative stem cell; ROC=receiver-operating characteristic.

**Table 2:** PSC marker expression in GC and MNGM

**PSC markers**	**Cancer and normal**	**Mean±s.d.**	***P-*value**
CD44	MNGM	0.5±2.5	*P*<0.001
	GC	64.3±88.3	
Musashi-1	MNGM	110.4±47.2	*P*<0.001
	GC	218.8±91.5	
CD133	MNGM	0.4±1.9	*P*<0.001
	GC	30.1±69.3	

Abbreviations: GC=gastric cancer; MNGM=matched normal gastric mucosa; PSC=putative stem cell.
