# Supplementary material for: Sequential expression of putative stem cell markers in gastric carcinogenesis
Source: Br J Cancer. 2011 Aug 9;105(5):658–65. doi: 10.1038/bjc.2011.287 (PMC3188930; doi:10.1038/bjc.2011.287)
Supplement: Supplementary Table S1 [file bjc2011287x1.pdf]

Supplementary Table S1: Co-relationships between putative stem cell marker expression and clinicopathologic features

	CD44			*Musashi-1*			*CD133*		
Clinicopathologic features	Analyzable tumour (N)	No. of positive expression (%)	P	Analyzable tumour (N)	No. of positive expression (%)	P	Analyzable tumour (N)	No. of positive expression (%)	p
**Age**									
<64	43	29 (67.4)	NS	42	34 (81.0)	NS	43	5 (11.6)	NS
>=64	68	46 (67.6)		60	49 (81.7)		68	14 (20.6)	
**Ethnicity**									
Chinese	98	65 (66.3)	**0.043**	90	73 (81.1)	NS	98	18 (18.4)	NS
Non-Chinese	12	11 (91.7)		11	9 (81.8)		12	1 (8.3)	
**Sex**									
Female	36	27 (75.0)	NS	33	27 (81.8)	NS	36	5 (13.9)	NS
Male	76	48 (63.2)		70	57 (81.4)		76	14 (18.4)	
**T**									
T1-T2	60	42 (70.0)	NS	53	38 (71.7)	**0.007**	60	10 (16.7)	NS
T3-T4	52	33 (63.5)		50	46 (92.0)		52	9 (17.3)	
**N**									
N0	34	22 (64.7)	NS	32	23 (71.9)	NS	34	7 (20.6)	NS
N1-N3	77	52 (67.5)		38	32 (84.2)		77	11 (14.3)	
**M**									
M0	102	68 (66.7)	NS	80	64 (80.0)	NS	102	17 (16.0)	NS
M1	10	7 (70.0)		10	9 (90.0)		10	2 (20.0)	
**Stage**									
I-II	55	38 (69.1)	NS	50	37 (74.0)	**0.047**	55	9 (16.4)	NS
III -IV	57	37 (64.9)		53	47 (88.7)		57	10 (17.5)	
**Tumour Grade**									
Well/moderate	29	16 (55.2)	**0.027**	30	23 (76.7)	NS	34	8 (23.5)	NS
Poor/undifferentiated	78	60 (76.9)		70	58 (82.3)		74	10 (13.5)	
**Histological type**									
Diffuse	54	44 (81.5)	**0.016**	52	45 (86.5)	NS	55	7 (12.7)	NS
Intestinal	51	31 (60.8)		50	38 (76.0)		56	12 (21.4)	
**H.pylori Co-Infection**									
Negative	28	18 (64.3)	NS	75	62 (82.7)	NS	81	15 (18.5)	NS
Positive	82	55 (67.1)		26	21 (80.8)		29	4 (13.8)	
**Perineural Invasion**									
No	60	39 (65.0)	NS	41	35 (85.4)	NS	60	7 (11.7)	NS
Yes	52	36 (69.2)		50	40 (80.0)		52	12 (23.1)	
**Lymph node Invasion**									
No	49	34 (69.4)	NS	37	29 (78.4)	NS	49	8 (16.3)	NS
Yes	63	41 (65.1)		60	49 (81.7)		63	11 (17.5)	

NS: not significant
