# Supplementary material for: Sequential expression of putative stem cell markers in gastric carcinogenesis
Source: Br J Cancer. 2011 Aug 9;105(5):658–65. doi: 10.1038/bjc.2011.287 (PMC3188930; doi:10.1038/bjc.2011.287)
Supplement: Supplementary Table S2 [file bjc2011287x2.pdf]

Supplementary Table S2: Clinicopathologic features of study cohort with neoadjuvant chemotherapy

Clinicopathologic features	Cases (N)
**Age**	
<55	4
>=55	4
**Ethnicity**	
Chinese	1
Non-Chinese	7
**Sex**	
Female	2
Male	6
**TNM Stage**	
II	3
III	5
**Tumour Grade**	
Moderate	1
Poor	7
**Histological type**	
Diffuse	6
Intestinal	2
**Perineural Invasion**	
No	5
Yes	3
**Lymph node Invasion**	
No	4
Yes	4
**Pathological response**	
No	4
Yes	4
