# Supplementary material for: Sequential expression of putative stem cell markers in gastric carcinogenesis
Source: Br J Cancer. 2011 Aug 9;105(5):658–65. doi: 10.1038/bjc.2011.287 (PMC3188930; doi:10.1038/bjc.2011.287)
Supplement: Supplementary Table S3 [file bjc2011287x3.pdf]

Supplementary Table S3: Dilutions used and manufacturers' information for antibodies used in immunohistochemistry (IHC) staining

Tumour markers	**Dilution for IHC**	**Clone**	**Manufacturer**
**CD44**	1:1000	SFF-304	Bender MedSystems, San Diego, CA
**Musashi-1**	1:600	AB5977	Millipore, Billerica, MA
**CD133**	1:20	AC133	MiltenyiBiotech, Auburn, CA
**Ki67**	1:1000	ab15580	Abcam, Cambridge, MA
**PCNA**	1:1000	NCL-PCNA	Novocasta, UK
